# Partial discharge localization in power transformer tanks using machine learning methods

**DOI:** 10.1038/s41598-024-62527-9

**Published:** 2024-05-23

**Authors:** Farzin Khodaveisi, Hamidreza Karami, Matin Zarei Karimpour, Marcos Rubinstein, Farhad Rachidi

**Affiliations:** 1 Department of Electrical Engineering, Bu-Ali Sina University, Hamedan, Iran; 2 University of Applied Sciences of Western Switzerland (HES-SO), 1400 Yverdon-Les-Bains, Switzerland; 3 Electrical Engineering Department, Hamedan University of Technology, Hamedan, Iran; 4 Electromagnetic Compatibility Laboratory, Swiss Federal Institute of Technology (EPFL), 1015 Lausanne, Switzerland

**Keywords:** Machine learning, Deep learning, Power energy system, Power transformers, Partial discharge localization, Conditional monitoring in power system, Electrical and electronic engineering, Power stations

## Abstract

This paper presents a comparison of machine learning (ML) methods used for three-dimensional localization of partial discharges (PD) in a power transformer tank. The study examines ML and deep learning (DL) methods, ranging from support vector machines (SVM) to more complex approaches like convolutional neural networks (CNN). Multiple case studies are considered, each with different attributes, including sensor position, frequency content of the PD signal, and size of the transformer tank. The paper focuses on predicting the PD location in three-dimensional space using single-sensor electric field measurements. Various aspects of each method are analyzed, such as the input signal, core methodology, correlation coefficient between the predicted location and the actual location, and root mean square error (RMSE). These features are discussed and compared across the different methods. The results indicate that the CNN model exhibits superior performance in terms of location accuracy among the methods considered.

## Introduction

Source localization has many applications in fields such as medicine, acoustics, electromagnetics, and lightning^1^. In the realm of electromagnetics, Partial discharges (PDs) are electrical breakdowns that occur within electrical insulations, such as those in transformers. Over time, PDs can lead to the complete breakdown of the insulation system, causing extensive damage to the transformer. PDs are a major contributor to the failure of power transformers, transmission lines, and gas insulation, among other components. Any malfunction in a power transformer can result in power outages and reduced reliability of electrical power networks. Therefore, the early detection and localization of PDs is crucial in order to prevent potential hazards and minimize further damage^2,3^.

PD localization techniques^2^ can be categorized into two groups: acoustic and electromagnetic. Acoustic detection and localization methods^2,4–7^ rely on detecting the sound waves emitted by PD sources. Compared to electromagnetic methods, acoustic methods are less sensitive to weak PDs and those that occur within the winding^7,8^. Acoustic sensors can be mounted on the external walls of the power transformer, making acoustic detection a non-invasive technique. Nevertheless, the acoustic signal may be contaminated by external acoustic environmental noise.

Electromagnetic localization based methods^2,4,7–10^ utilize electromagnetic waves emitted by PD sources. The detection methods that employ ultra high frequency (UHF) radiation are particularly sensitive to weak PDs occurring within the winding. Moreover, UHF measurements are commonly electromagnetically shielded by grounding the transformer tank to mitigate external disturbances such as corona and environmental noise^11^.

Classical acoustic and electromagnetic three-dimensional localization methods rely on the Time Difference of Arrival (TDoA) of signals. However, these methods are highly sensitive to noise due to the need for precise determination of the onset time of the arriving signals^11^. Moreover, they require a minimum of four time-synchronized sensors as well as direct propagation paths from the PD sources to the multiple sensors to operate effectively. In the case of acoustic methods, reasonable accuracy can be achieved by implementing appropriate signal processing techniques. However, TDoA-based electromagnetic methods encounter inaccuracies caused by inhomogeneities and scattering within transformers.

Recently, a novel approach based on time reversal has been proposed in the electromagnetic and acoustic regimes. This approach can localize the sources of partial discharges inside a transformer using only a single sensor. In comparison to the conventional TDoA method, the time reversal-based method demonstrates robustness to noise in the experimental signals. Moreover, it remains effective even in the presence of obstacles that obstruct the direct line of sight between the sensor and the PD source. The technique requires a model of the transformer tank to carry out the backward propagation stage^12^.

The creation of a PD localization system demands a significant level of accuracy, sensitivity, and robustness. These qualities have been essential for power grid operators and installers over the past decades. Traditionally, PD diagnostics primarily rely on features extracted through conventional techniques such as statistical analysis, and time–frequency analysis. Simple threshold values are then computed to make decisions^11^. Advanced signal processing techniques like the discrete wavelet transform (DWT) are employed to extract more sophisticated and powerful features, while conventional machine learning (ML) methods, including Back-Propagation Neural Networks (BPNN), support vector machines (SVM), and fuzzy inference systems (FIS), are gradually utilized for classification and regression tasks^11^. In recent times, with advancements in computing and information technologies, deep learning (DL) has gained significant attention as a subset of ML for intelligent PD diagnostics^13^.

Table S1, based on the review of^13^ and including articles published since 2021, presents an overview of papers that utilize ML methods for PD diagnosis. The studies predominantly focus on detection, pattern recognition, and classification, as shown in the table. However, only about 12.7% of these studies address the problem of localization. In terms of applications, only 16.50% of the studies are focused on transformers, while others examine PDs in gas-insulated transmission lines (GIL), gas-insulated switchgear (GIS), high-voltage cables, and electrical equipment. One of the primary reasons for the limited attention given to PD localization is its inherent difficulty compared to detection and classification. Notably, the CNN model has garnered researchers’ attention due to its exceptional performance in signal and image processing, as depicted in Table S1. For PD localization, the bagging-kernel extreme learning machine (Bagging-KELM)^14^ achieves the best result in GIL, with an average error of 0.93 cm. Neural networks, bagging techniques, and SVMs are the most frequently employed models in PD localization studies. It should be noted that all the methods presented in Table S1 have been individually investigated, considering various configurations and scenarios. This diversity in approaches has made the task of comparison quite challenging. The aim of this paper is to provide a comprehensive comparison of these models within specific and well-defined scenarios. In particular, several well-known ML-based methods are investigated for the three-dimensional localization of partial discharges inside a power transformer tank. ML and DL methods frequently used in recent articles on PDs are examined, considering their compatibility for regression problems. Multiple case studies involving various attributes are presented. These attributes encompass sensor positioning, number of sensors, frequency content of the PD signal, and the size of the transformer tank. The PD location in the three-dimensional space is determined using single-sensor electric field measurements for all case studies, except for one case study in which three sensors are considered. The features of each method, such as input signal, core methodology, correlation coefficient of predicted location with the real location, and root mean square error (RMSE) analysis, are discussed and compared.

The novelty of the paper lies in the three-dimensional localization of PD sources within a power transformer tank using only a single sensor, achieved through ML and DL techniques. These techniques include BPNN, CNN, SVR, and XGBoost methods. The paper also provides a comprehensive comparison of the performance of each method in localizing the PD sources.

The remainder of the paper is organized as follows: “Case studies” presents the data generation, “Data preprocessing” illustrates the data preprocessing procedure, and in “Machine learning methods”, explanations for each model are provided. “Results and discussion” focuses on the model comparison and presents the results. Finally, in “Conclusion”, concluding remarks are provided.

## Case studies

**Figure 1:**
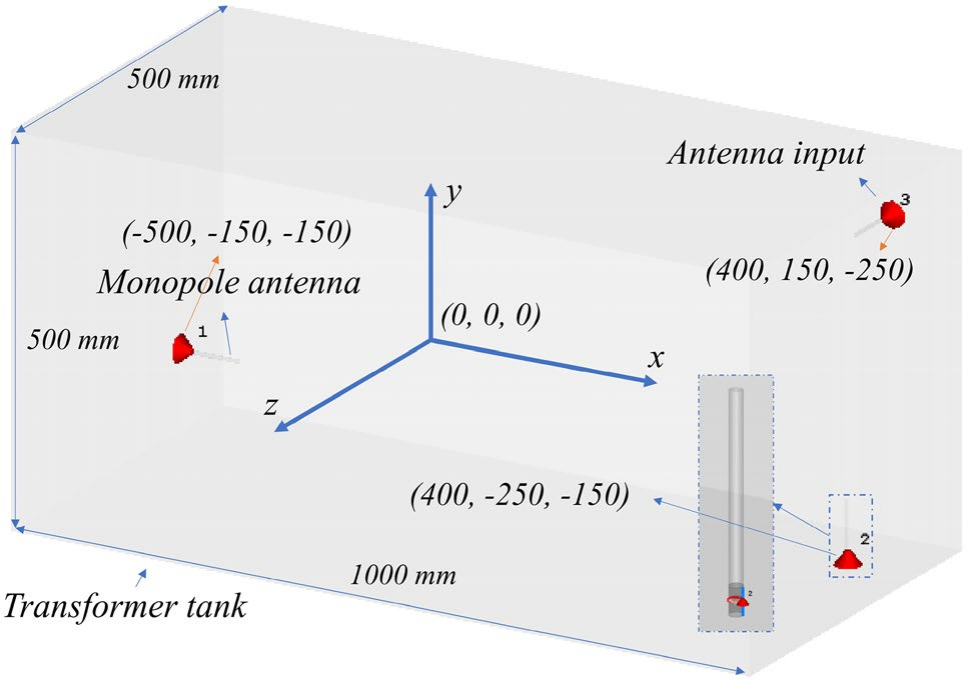
The transformer tank including three identical monopole antennas representing sensors aligned in three different axes. The inset is a zoom of antenna 2.

This section presents the various case studies considered in the analysis. All the case studies have been simulated using microwave studio (CST) software. The geometry of the transformer tank is illustrated in Fig. 1. The origin of the coordinate system is located at the center of the transformer tank. For simplicity, the study does not include the windings and ferromagnetic cores. The transformer tank is made of steel with a conductivity σ of 7.69e6 S/m. The volume of the transformer tank, as shown in Fig. 1, is 1000 × 500 × 500 mm^3^. The thickness of the tank walls is 10 mm. In the study, the PD sources are modeled as small dipole antennas with a length of 10 mm, excited by a Gaussian pulse. The figure does not depict the dipole antenna used to model the PD source. Different orientations of the PDs are considered in the considered case studies. Please refer to Table 1 for further details.

**Table 1:** Five different case studies are discussed in the paper.

Case study	Tank dimension (mm^3^)	PD orientation	Antenna polarization^1^	Bandwidth (GHZ)	Number of samples
CS#1	1000 × 500 × 500	y-axis	x-axis	0.5–3	600
CS#2	1000 × 500 × 500	y-axis	y-axis	0.5–3	1000
CS#3	1000 × 500 × 500	Random	x-, y-, or/and z-axis	0.5−*f*_max_^2^	2000
CS#4	1000 × 1000 × 500	Random	x-, y-, or z-axis	0.5−*f*_max_	1000
CS#5	2000 × 1000 × 1000	Random	x-, y-, or z-axis	0.5−*f*_max_	1000

^1^The three monopole antenna feeds are located, respectively, at positions (x = −500 mm, y = −150 mm, z = −150 mm), (x = 400 mm, y = −250 mm, z = −150 mm), and (x = 400 mm, y = 150 mm, z = −250 mm). ^2^*f*_max_ is randomly selected between 1.0 to 3.0 GHz.

The emitted fields from the PD sources are detected by three different sensors, represented by monopole antennas, as shown in Fig. 1. The length and radius of the monopole antennas are 67.8 and 2.5 mm, respectively. The red cones in Fig. 1 depict the antenna inputs. Five different case studies are discussed in the paper, as shown in Table 1. The description of each case study is presented in the subsequent subsections.

### Case study #1

In the first case study, a single monopole antenna placed along the x-axis is employed to receive the PD signal (see Fig. 1 and Table 1). The coordinates of the monopole antenna are (x = −500 mm, y = −150 mm, z = −150 mm). The PD is modeled as a 10 mm y-polarized dipole antenna positioned randomly within the transformer tank of Fig. 1. The PD signal in the simulation is a Gaussian pulse with a frequency bandwidth of 0.5–3 GHz, see Table 1. A total of 600 Monte-Carlo simulations were conducted for this case study. The location of the PD source within the transformer tank was randomly selected using a uniform probability distribution function for each direction.

### Case study #2

The second case study is similar to CS#1, except for the location and polarization of the monopole antenna used to receive the PD signals. In CS#2, a y-axis polarized monopole antenna is employed as the receiving sensor, situated at coordinates (x = −400 mm, y = −240 mm, z = −150 mm). This case study utilizes 1000 Monte-Carlo simulations, see Table 1.

### Case study #3

In the third case study, the location of the PD sources is randomly selected inside the transformer tank using a uniform probability function, similar to case study #1. However, unlike the previous case studies, the PD source polarization is arbitrary. To achieve this, three new variables are introduced to indicate the rotation angles along the x, y, and z axes. These three angles are selected using a uniform probability distribution function in the range of 0–360 degrees. The three dipole antennas are used to record the electric fields emitted by the PD source. However, even though the fields are captured by all three antennas, each at a different location and with a different polarization, the captured signals from the monopole antennas are considered separately. In other words, for the purpose of localizing the PD source, the electric field components are utilized individually. The locations of the receiving antenna can be found in Fig. 1.

The PD signal used in this case study is a Gaussian pulse with a frequency bandwidth covering the range of 0.5 to fmax GHz. The value of fmax is randomly selected using a uniform probability distribution function between 1 and 3 GHz. A total of 1000 Monte Carlo simulations were conducted for this case study to ensure a consistent number of instances and maintain uniform fairness across all case studies. Additionally, another 1000 samples are planned for consideration in the triple sensor case study.

### Case study #4

The fourth case study is similar to the previous case study (CS#3), but it uses a larger transformer tank size: 1000 × 1000 × 500 mm^3^, which is twice as long in the y-axis direction compared to the tank size used in the previous three cases. The locations of the monopole antennas used in this study are, respectively, at positions (x = −500 mm, y = −400 mm, z = −150 mm), (x = 400 mm, y = −500 mm, z = −150 mm), and (x = 400 mm, y = 400 mm, z = −250 mm).

### Case study #5

The transformer tank size used in this last case study is 2000 × 1000 × 1000 mm^3^, which means that the length of the tank sides along the axes is twice that of the tank used in CS#1 to CS#3. The monopole antennas used in this study are, respectively, at positions (x = −500 mm, y = −400 mm, z = −150 mm), (x = 400 mm, y = −500 mm, z = −150 mm), and (x = 400 mm, y = 400 mm, z = −250 mm).

## Data preprocessing

Data preprocessing is a crucial step in signal processing that plays a vital role in achieving accurate and efficient solutions with minimal complexity. Moreover, preliminary experiments have demonstrated the necessity of preprocessing of both the PD signals and actual labels (locations). In this study, data preprocessing consists of five key steps: cut-off, normalization, resampling, label shifting, and train-test dataset splitting.

### Cut off

The first stage of preprocessing involves trimming a specific duration of time from all signal instances. This step is crucial in simulations because the wave maintains a constant speed, and the onset time provides information about the location of the PD (partial discharge) in the radial direction. However, this approach can lead to unfair predictions when compared to practical tests. To achieve a more robust model, it is beneficial to implement this preprocessing step.

Specifically, the duration of the signal is cut down to 40 ns by trimming the beginning and the end as follows: A starting threshold is defined as the time at which the signal begins to fluctuate more than 0.001 V in amplitude. This threshold is denoted as *t*, representing the starting time. Any data prior to this threshold is discarded. The remaining time duration (duration of the signal–*t*) is then trimmed from the end of the signal so that the total duration is 40 ns. However, since the initial sample rates of the instances differ, they will have varying numbers of samples.

### Normalization

The next step in preprocessing is normalization, which aims to expedite the training of the model. To achieve this, the entire signal is divided by the absolute value of the maximum signal amplitude, which can vary significantly for PD signals in the database. Consequently, the output signal is constrained to fluctuate between −1 and 1.

### Resampling

The simulated signals generated using the CST-MWS software have varying sampling rates due to the implementation of the finite integration technique during the simulations. Consequently, these signals have different numbers of samples. To ensure consistency in the input shape of the model, resampling becomes a crucial step, aiming to achieve an equal number of samples for all signals. In this paper, the down-sampling procedure is based on polyphase filtering, which offers computationally efficient resampling and filtering capabilities with high accuracy when applied to signals with defined sample rates.

Based on the conducted experiments, the best performance was observed when using 400, 800, and 1200 samples as the number of input features for the model for values ranging between 50 and 4800 for SVR. The number of samples does not have a significant effect on XGBoost performance. Consequently, a value of 400 samples was selected. Increasing the number of samples beyond this value would not significantly enhance accuracy but would significantly prolong the model training process. Additionally, polyphase filtering has proven to be a suitable approach, preserving over 98% of the signal content, as indicated by the computed correlation coefficient between the original and resampled signals.

**Figure 2:**
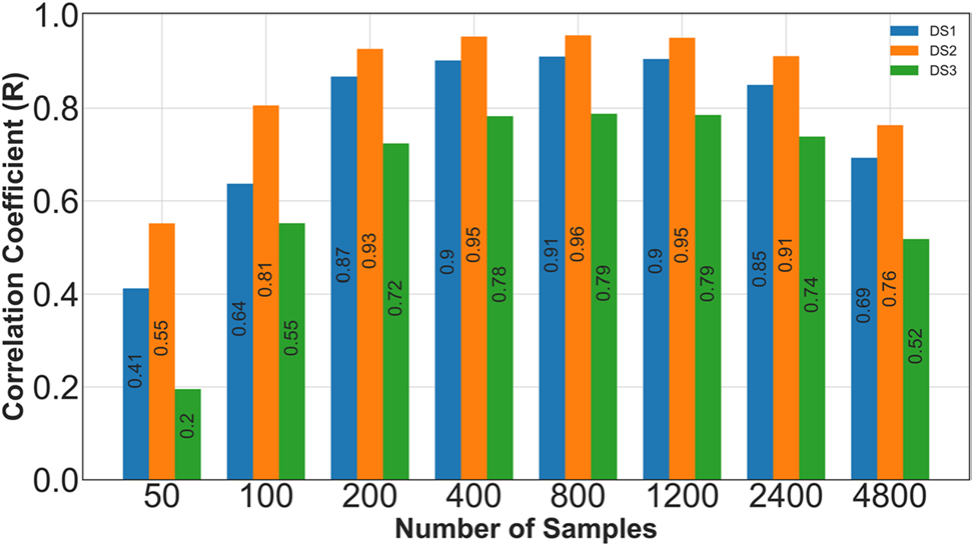
The variation of the R metric versus the number of samples for three separate datasets (CS#1, CS#2, and CS#3) along the y-axis using the SVR method.

In Fig. 2, the effect of sample rates ranging from 50 to 4800 is depicted across three separate databases (used for CS#1, CS#2, and CS#3) for the x, y, and z directions (horizontal axis). The coefficient of determination (R) for predicted locations by the Support Vector Regression (SVR) model is shown on the vertical axis. The best result is obtained with a number of samples ranging from 400 to 1200. Therefore, signals with a sample rate of 400 are used throughout the paper to reduce computational tasks.

### Shifting labels (location of PD sources)

Since labels are ranged from negative to positive numbers, which correspond to the location of the PD source inside the cavity along the x-, y-, and z-axis, the model might not be able to distinguish the sign of numbers during both the training and evaluation stages. The solution used here involves shifting all the labels to positive regions, thereby yielding a more resilient model. Nonetheless, the amount of shifting may vary depending on the specific tank shape in each case study.

### Splitting training and test dataset

Before training, the datasets are divided into train and test data, with 80% of the dataset used for training and 20% for testing. To ensure a fair comparison of results between models, the training dataset is randomly shuffled using a seed number of 11. This procedure guarantees that each partition undergoes a complete pattern randomization.

## Machine learning methods

A flowchart depicting the ML and DL-based approaches proposed in the paper is presented in Figure S1 in the Supplementary Information. The initial step involves data collection, which is simulated using CST-MWS software. Once the data is collected, it needs to undergo preprocessing before being fed into the models. During preprocessing, the data is initially trimmed and then normalized to fall within the range of 1 to −1. Following normalization, the data is resampled to consist of 400 samples. Given that the labels span from negative to positive numbers, representing the PD source’s location within the cavity along the x-, y-, and z-axis, the location labels' origin is shifted to ensure all labels are positive.

After the data is prepared for model input, a range of models is assessed to identify the one with the highest accuracy. Subsequently, these models are trained using 80% of the preprocessed data and evaluated using the remaining preprocessed data for testing purposes. Finally, the model that performs the best is selected as the optimal choice.

Four frequent models have been chosen from those in Table S1: Support Vector Machine (SVM), neural networks (NN), convolution neural networks (CNN) and XGBoost which encompasses boosting methods.

Each model used in this paper has gone through a grid search for hyperparameters. The grid search condition is slightly different depending on each model architecture. All ML methods used in this paper have the same input: a 1D preprocessed PD signal in the time domain with 400 samples, except for three sensor case study (see “Three sensors”, for which 1200 samples were used. To assess the degree of association between two variables, correlation coefficients are used.

### Support vector regression

A simple linear support vector machine (SVM) classifier operates by drawing a straight line between two classes. This means that all the data points on one side of the line will be classified as one category, while the data points on the other side will be assigned to a different category. As a result, there are numerous possible lines to select from.

Support vector regression (SVR) applies the same principle as SVM, but it is used for regression problems. SVR is a widely used algorithm with various applications^15^. To optimize SVR, a grid search is performed on the gamma, regularization parameter, and kernel. The best outcome was achieved by setting the gamma value to 0.01, the regularization parameter to 1000 (where the strength of regularization is inversely proportional) and employing the radial basis function (“RBF”) as the kernel. Since SVR does not inherently support multidimensional regression, the multi-target regression strategy is employed to expand its capabilities, fitting one regressor per target.

### XGBoost

A gradient boosting decision tree (GBDT) is an ensemble learning algorithm, similar to random forest, used for both classification and regression tasks. Ensemble learning algorithms combine multiple machine learning algorithms to obtain improved models. XGBoost is an example of a parallel tree boosting algorithm^16^ and it is implemented using the XGBoost library. In this case, default hyperparameters are used as the model performance does not improve after grid search. Additionally, XGBoost also supports multi-target regression strategy.

### back-propagation neural network (BPNN)

**Figure 3:**
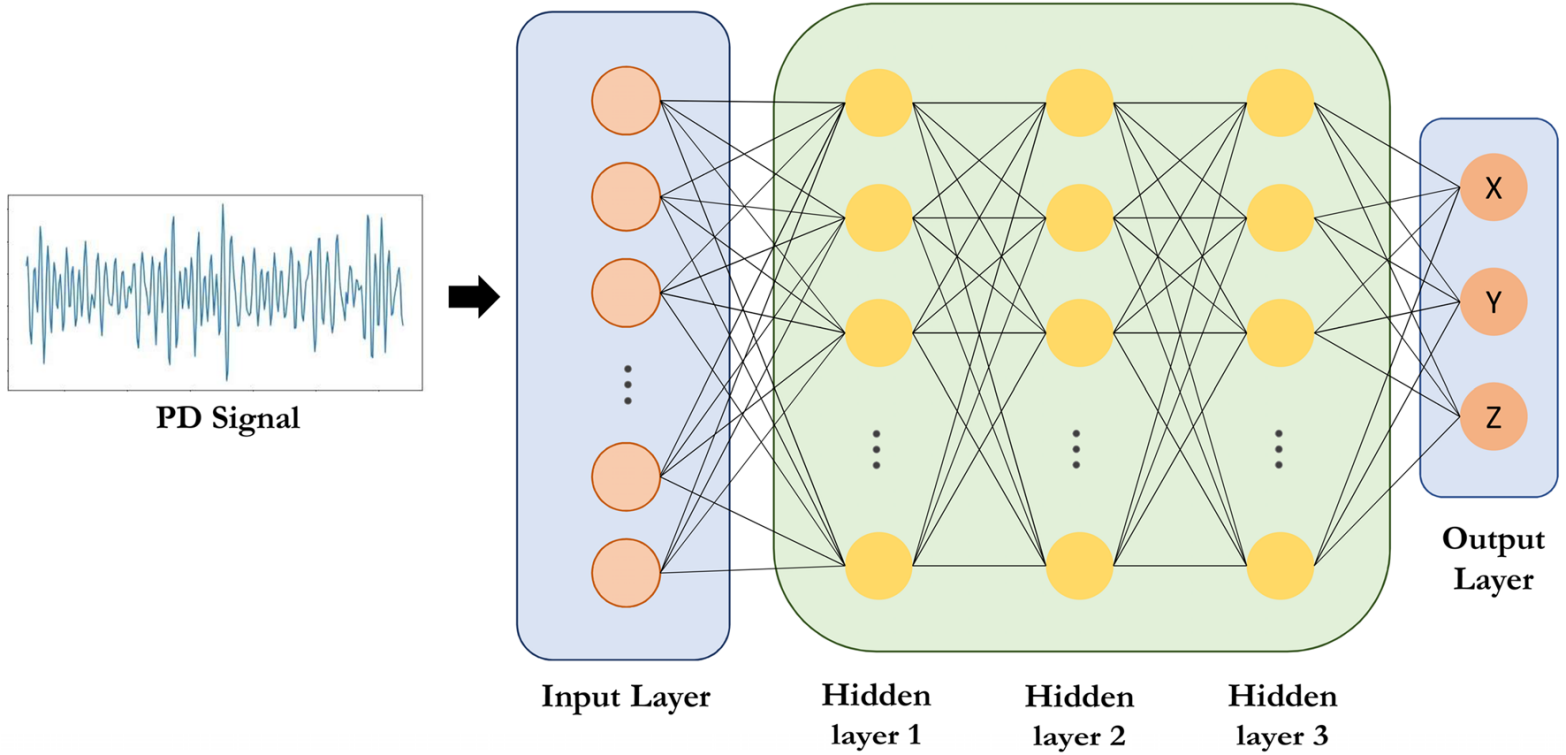
Architecture of BPNN model. The first layer is the input layer, each fully connected layer has 512 units, and the output layer estimates the x, y and z coordinates of the PD source.

DL has made significant progress in various applications. One of the first DL models that has been extensively examined is the Backpropagation Neural Network (BPNN). The BPNN consists of multiple layers, with each layer containing a number of neurons that adapt complex functions through a series of nonlinear transformations. The architecture of this model is illustrated in Fig. 3. It comprises three main parts: the input layer, hidden layers, and output layer.

The input layer serves as a simple fully connected layer that feeds into the hidden layers. The hidden layers consist of three dense layers, each containing 512 units with the rectified linear unit (ReLU) activation function. On the other hand, the output layer is another dense layer with three units representing the 3D source location. To optimize the model, the Nadam optimizer^17^ is used, and the learning rate gradually decreases from 0.1 to 0.001.

### Convolutional neural network (CNN)

**Figure 4:**
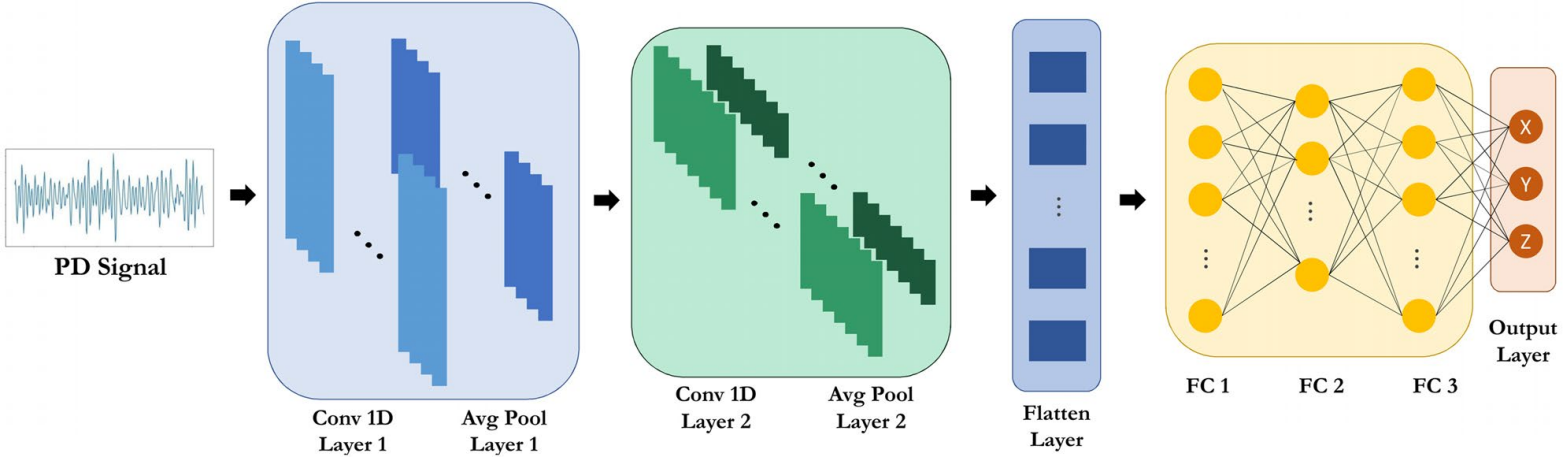
Architecture of the CNN model. The input layer consists of 400 nodes. Layer 1 is a 1D CNN layer with dimensions (394, 64) followed by an average pooling layer with dimensions (98, 64). Layer 2 is another 1D CNN layer with dimensions (89, 256) followed by an average pooling layer with dimensions (44, 256). The first fully connected (FC) layer has 512 units, the second FC layer has 256 units, and the third FC layer has 512 units. The output of the model represents the x, y, and z coordinates of PD.

A convolutional neural network (CNN) operates in a similar manner to conventional fully connected multilayer perceptron neural networks, but with additional convolutional layers positioned at the front of the network^18^. The model considered in this study is the 1D CNN model^19^. This particular model yielded the best results, as indicated in Table 2. In comparison to the back-propagation neural network (BPNN), the CNN 1D model is more complex, which leads to higher computational cost but also improved accuracy. All layers in the model employ the rectified linear unit (ReLU) activation function, and the optimizer used is similar to that of the back propagation neural network (BPNN). For a comprehensive representation of the model’s architecture, please refer to Fig. 4.

**Table 2:** RMSE in mm and correlation coefficient (R) for each model and case study. For CS#3, three different directions corresponding to the receiving antenna are listed as X, Y, and Z. The origin of the coordinate system is at the center of the transformer tank.

Models case studies			SVR	XGBOOST	BPNN	CNN
CS#1	R	x	0.94	0.88	0.94	0.99
		y	0.91	0.91	0.91	0.97
		z	0.84	0.73	0.86	0.94
	RMSE		74.46	95.57	73.22	39.89
CS#2	R	x	0.94	0.89	0.94	0.99
		y	0.96	0.84	0.95	0.98
		z	0.94	0.88	0.89	0.97
	RMSE		58.76	83.62	60.98	27.04
CS#3 X	R	x	0.92	0.83	0.9	0.95
		y	0.42	0.36	0.5	0.56
		z	0.4	0.32	0.5	0.59
	RMSE		106.46	122.51	102.42	89.44
CS#3 Y	R	x	0.87	0.81	0.87	0.96
		y	0.77	0.49	0.74	0.83
		z	0.69	0.55	0.65	0.8
	RMSE		97.38	117.71	100.18	67.14
CS#3 Z	R	x	0.86	0.81	0.82	0.93
		y	0.75	0.58	0.71	0.81
		z	0.69	0.3	0.65	0.79
	RMSE		100.46	119.82	109.2	75.7

CNN-based methods automatically identify and utilize hierarchical features in signals received by sensors. In CNN-based methods, multiple layers of convolutional filters are applied to the signal, progressively obtaining higher-level features. This is crucial for localizing partial discharges, where the spatial information of the source is encoded in the signal. Other methods like SVM and XGBoost rely on global features extracted from the signal in partial discharge localization applications. It should be noted that in other applications, feature engineering can improve the performance by selecting the best features to achieve better results. For example, SVM-based approaches excel in classification tasks where feature boundaries can be distinctly defined in a high-dimensional space but do not inherently extract features from complex patterns like images. Therefore, through the use of convolutional layers and pooling operations, CNNs can capture spatial hierarchies and dependencies between different parts of the data, such as the location and spread of discharge patterns within a transformer tank.

## Results and discussion

All models are evaluated based on their performance measured by the root mean square error (RMSE) and correlation coefficient (R) criteria in each coordinate for all case studies. The Pearson correlation coefficient^20^ is a numerical measure that determines the linear correlation between measured values and values simulated by the model, with an optimal value of 1.

1 $$R_{ij} = \frac{{C_{ij} }}{{\sqrt {C_{ii}C_{jj} } }},$$

In Eq. (1), the variable *i* represents the actual location, while *j* represents the predicted location in the same direction, such as the y direction. The parameter *C*_ij_ denotes the covariance between *i* and *j*, and *C*_ii_ represents the standard deviation of *i*.

One way to evaluate the goodness of fit of a regression model to a dataset is by calculating the Root Mean Square Error (RMSE). RMSE is a metric that measures the distance between the predicted values from the model and the actual values in the dataset. A lower RMSE indicates a better fit of the model to the dataset. The formal definition of RMSE is as follows:

2 $$RMSE = \sqrt {\mathop \sum \limits_{i = 1}^{n} \frac{{\left( { \hat{x}_{l} - x_{i} } \right)^{2} + \left( {\hat{y}_{l} - y_{i} } \right)^{2} + \left( {\hat{z}_{l} - z_{i} } \right)^{2} }}{n}} ,$$

where, ${\widehat{x}}_{l}$, ${\widehat{y}}_{l}$, and ${\widehat{z}}_{l}$ are predicted values, *x*_*i*_, *y*_*i*_, and *zi* are observed values respectively. The quantity *n* is the number of samples.

The constructed models were trained and tested using eightfold cross-validation. However, for all the results presented in this paper, the seed number 11 was used to split the training and test datasets. The implementation was done using the Python programming language, and the models were trained and evaluated on a computer with an NVIDIA GeForce GTX 1660 TI and 4 GB of graphics memory. To facilitate further research, all codes and datasets used in this study have been made available on GitHub. (https://github.com/Farzinkh/Partial_Discharge.)

### Single sensor

Table 2 presents the R metric (corresponding to the correlation coefficient of the PD source estimation) and the RMSE value (corresponding to the three-dimensional localization error) for four different models: SVR, XGBoost, BPNN, and CNN. The first main column provides experiment details, including the case study number (refer to Table 1), and displays the R metric or the RMSE. The second to fifth columns present the results for the SVR, XGBoost, BPNN, and CNN models, respectively. For instance, the shaded row in Table 2 represents the R metric for the z-coordinate of all the different models in the first case study (CS#1).

**Figure 5:**
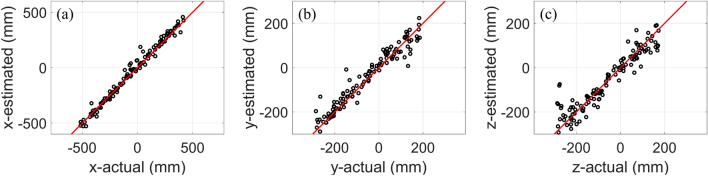
The CNN model’s estimated location compared to the actual location of PD sources for CS#1: (**a**) x-coordinate, (**b**) y-coordinate, and (**c**) z-coordinate. The number of instances for all the curves is 120.

In CS#1, the CNN model performs the best, with accuracies of 0.99, 0.97, and 0.94 for the x, y, and z-coordinates, respectively. The RMSE is 39.89 mm, which is considered excellent for partial discharge applications. In this case study, the receiving antenna is oriented along the x-axis, and the PD source polarization is along the y-axis. The second-best model is BPNN, which achieves accuracies of 0.94, 0.96, and 0.80 for the x, y, and z-coordinates, respectively. SVR exhibits similar performance to BPNN, with a slight reduction (2 percent) in the estimation accuracy along the z-coordinate. Finally, XGBoost achieves accuracies of 0.8, 0.91, and 0.73 for the x, y, and z-coordinates, respectively. The localization error averages for SVR, XGBoost, BPNN, and CNN are 74.46, 95.57, 73.22, and 39.89 mm, respectively. Figure 5 (a) (b), and (c) show the evaluation curves (the estimated versus the actual location of the PD source) for the CNN method for the x, y, and z coordinates in CS#1.

**Figure 6:**
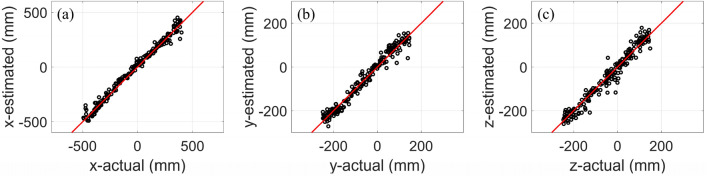
The CNN model’s estimated location compared to the actual location of PD sources for CS#2: (**a**) x-coordinate, (**b**) y-coordinate, and (**c**) z-coordinate. The number of instances for all the curves is 196.

In CS#2, the performance of the CNN method is better than in other models, similar to the previous case study. It can estimate the PD source location with accuracies of 0.99, 0.98, and 0.97 for the x, y, and z-coordinates, respectively. In contrast to CS#1, the SVR performs slightly better than the BPNN method. The accuracies of SVR and BPNN are (0.94, 0.96, 0.94) and (0.94, 0.95, 0.89), respectively, with each parenthesis representing the x, y, and z coordinates. Finally, the XGBoost method presents the worst results in terms of accuracy in estimating the PD source. Its accuracy is lower than 0.89 for all coordinates. In CS#2, both the antenna direction and PD polarization are along the y-axis. The localization error averages for SVR, XGBoost, BPNN, and CNN are 58.76, 83.62, 60.98, and 27.04 mm, respectively. Figure 6 (a), (b), and (c) show the evaluation curves (the estimated versus the actual location of the PD source) for the CNN method for the x, y, and z coordinates in CS#2.

**Figure 7:**
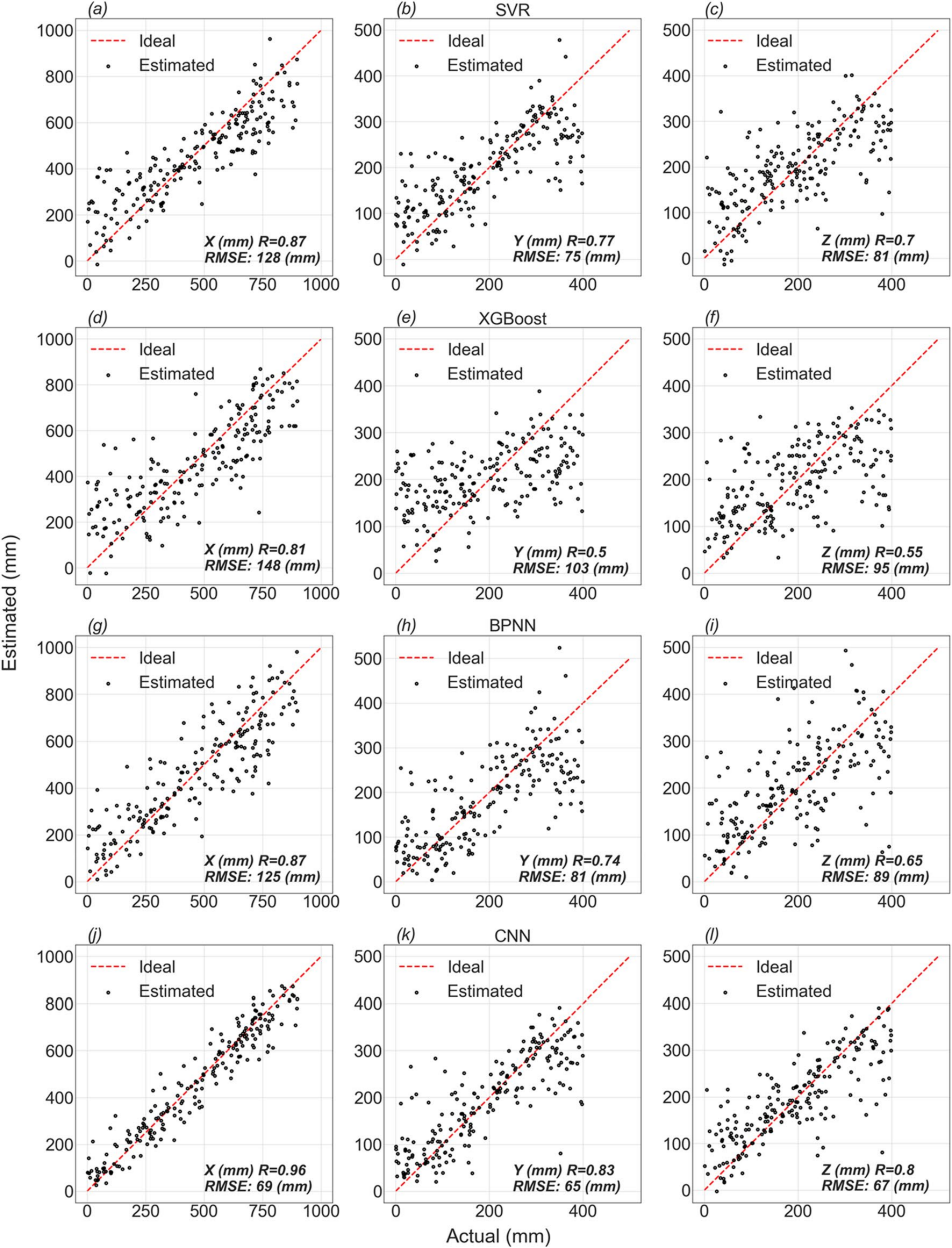
Evaluation curves for (**a**–**c**) SVR, (**d**–**f**) XGBoost, (**g**–**i**) BPNN, and (**j**–**l**) CNN (the y direction is considered for the receiving antenna) methods. The number of instances for all the curves is 200.

The last three main rows in Table 2 are devoted to CS#3. Unlike the two previous case studies (i.e., CS#1 and CS#2), the performance of all models is reduced. This is because in CS#3, the polarization of the PD source is randomly changed in the simulation. Generally, the CNN method performs better than the other methods, similar to the previous case studies. In CS#3, when the receiving antenna along the x-axis is used, the performance of BPNN is better than SVR; otherwise, SVR outperforms the BPNN method. In this case study, like the previous ones, the performance of XGBoost is the worst. The evaluation curves for all four models are shown in Fig. 7. It can be observed from the figure that the performance of the CNN method is superior to that of the other methods. The CNN exhibits higher accuracy for the x-coordinate compared to the y and z coordinates, as indicated in Table 2. According to Table 2 and Fig. 7, it is evident that, across all techniques and case studies (especially for CS#3), the accuracy of PD source estimation yields better results for the x-coordinate. To investigate the reason behind this observation, CS#4 and CS#5 were employed.

**Figure 8:**
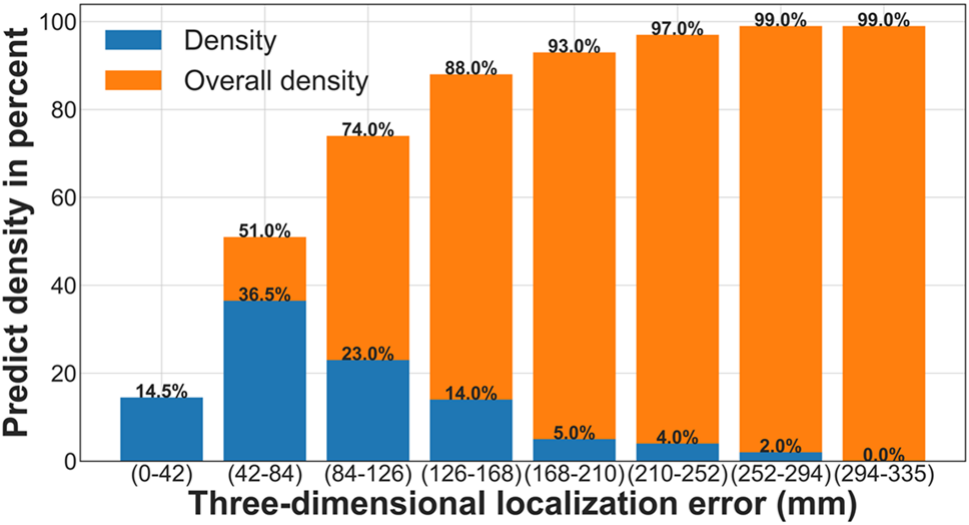
The density of three-dimensional localization error obtained from the CNN model. The blue bars represent the local density, while the yellow bars represent the overall density.

Observe the antenna oriented along the y axis in CS#3 (forth column of Table 2). In this case, the minimum and maximum localization errors are 12.1 and 347.54 mm, respectively, and the mean value is 98.09 mm. Figure 8 displays the density of the three-dimensional localization error obtained from the CNN model on the test dataset. For better insight, all predicted errors are classified in Fig. 8 into eight 42 mm bins, starting with zero and ending with the maximum error. The blue bars represent the local density in each stage, while the yellow bars represent the overall density. According to this figure, 88% of PD source localizations have errors less than 168 mm (lower than 17 cm), which validates the relatively accurate nature of this model in predicting locations of PD sources.

**Figure 9:**
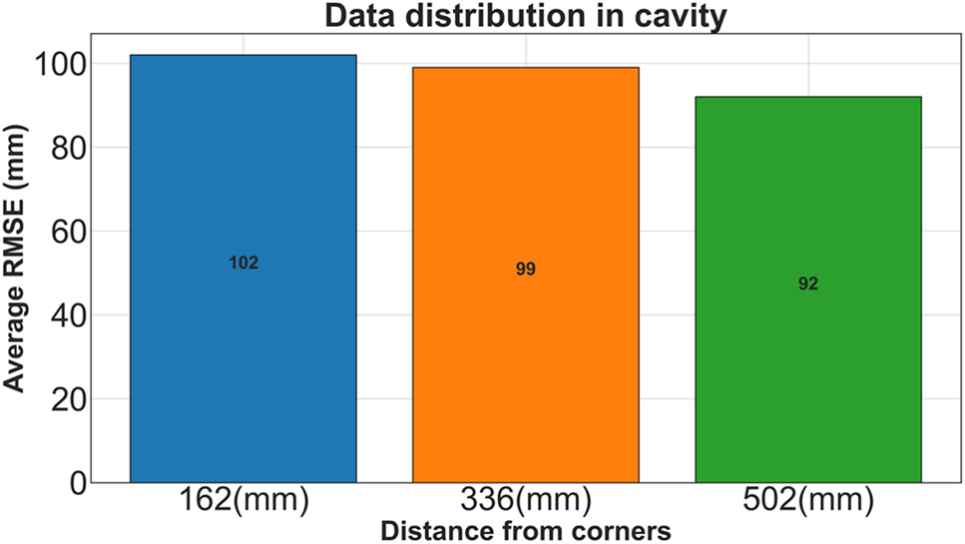
The RMSE versus the location of the PD source inside the transformer for CNN method in CS#3 in ranges less than 162, between 162 and 336 and more than 336.

To investigate the effects of the PD’s location on the obtained results, Fig. 9 presents the RMSE of the CNN model results for CS#3 with the y-direction receiving antenna. The transformer tank is divided into three sections based on the distance between each section and the corner of the transformer tank. The vertical axis represents the RMSE for each section. It can be observed that the CNN method can accurately estimate the PD’s location anywhere inside the tank, as the localization error associated with the PD's location in the CNN method is negligible.

### Three sensors

**Figure 10:**
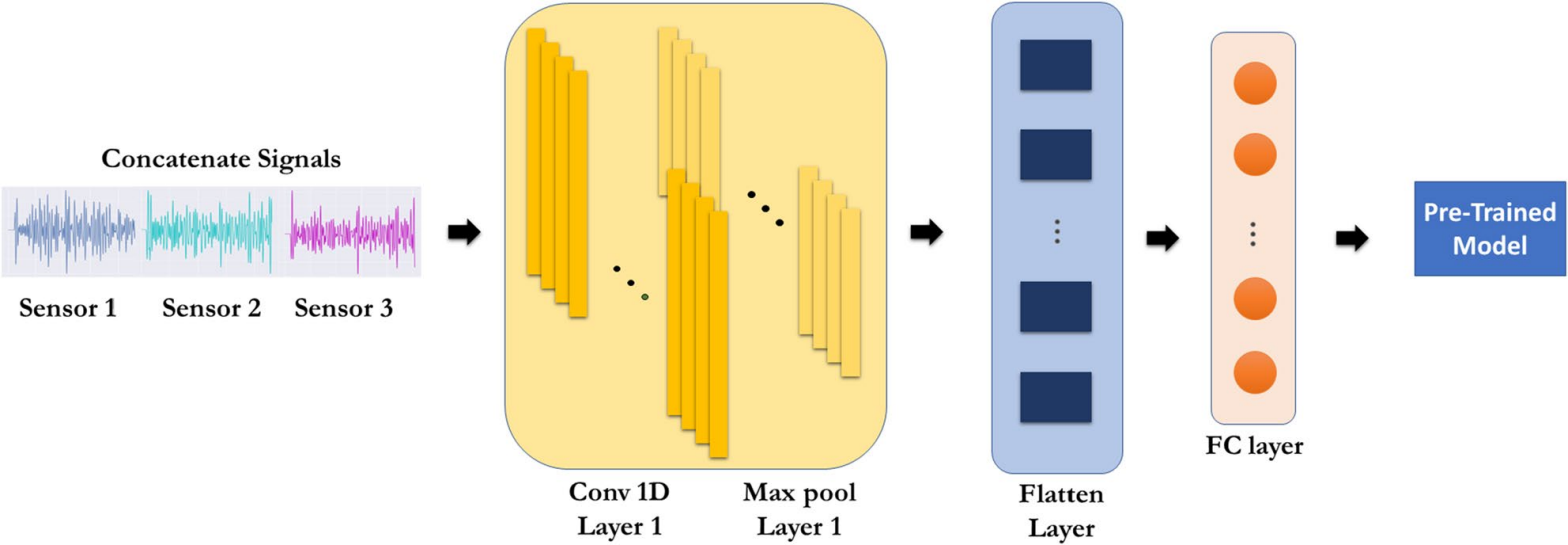
Architecture of the CNN model to utilize transfer learning techniques.

A possible approach to increase the accuracy of the model is to increase the number of sensors, as increasing the number of samples for each signal does not provide any significant advantage (see Fig. 2). Using three separate sensors in different directions is beneficial when dealing with a variety of PD frequencies (ranging from 0.5 to 3 GHz). The procedure becomes slightly more complex in terms of the model architecture, as shown in Fig. 10. Based on the conducted experiments, since simple preprocessing methods for merging PD signals like summation, subtraction, and averaging as feature extraction on three signals (each containing 400 samples) in the element-wise procedure do not improve the accuracy of the model, a more advanced method was required. One solution for achieving high model accuracy is by employing CNN models once again as feature extraction layers to achieve a 400-sample signal which is the desired input shape for the base model and utilizes the transfer learning technique. According to the figure, a solution for achieving high model accuracy in merging the PD preprocessed signals (each containing 400 samples) is to employ CNN models once again and to utilize transfer learning techniques. The architecture used to adapt a signal with 400 samples for input into the preceding model (the base model) to include a CNN 1D layer (1137,10), a Max pooling layer (162,10), and an FC layer with 400 units as embedding layer. These extra layers are added before the base model. It is important to note that the preceding model plays a vital role and was specifically trained for one sensor operating within the 0 to 3 GHz PD frequency range.

For this experiment, two scenarios are considered. The datasets of CS#3 are divided into two equal parts, each with a length of 1000 samples. The first scenario involves training the base model on CS#3 (Part I) and CS#3 (Part II) for 3-sensor transfer learning. The second scenario reverses the order of the datasets. The results of these two experiments are presented in Table 3. In the first scenario, the RMSE error decreases from 67.14 to 46.13 mm for the single-sensor and the three-sensor CNN models, respectively, leading to a 31.2% improvement. In the second scenario, the RMSE error decreases from 84.2 to 61.85 mm for the single and the three-sensor CNN models, respectively, leading to a 22.35% improvement. According to these records, the use of three sensors lead to an improvement in the accuracy of about 26%.

**Table 3:** RMSE in mm and correlation coefficient (R) for the CNN method for CS#3.

Pre-trained model dataset	Direction	Accuracy (R)	
		R	RMSE (mm)
CS#3 (Part I)	x	0.9821	46.1367
	y	0.9262	
	z	0.9169	
CS#3 (Part II)	x	0.9679	61.8552
	y	0.8327	
	z	0.8668	

**Figure 11:**
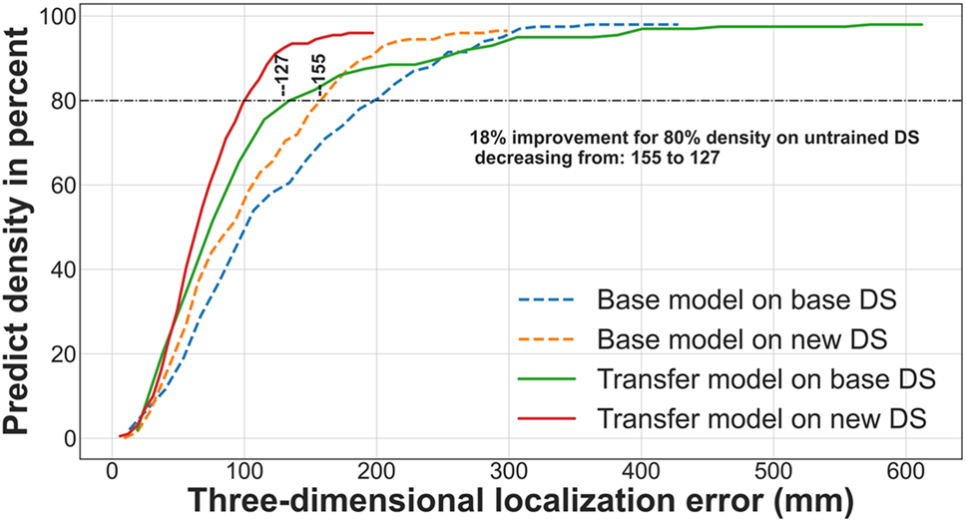
The density of three-dimensional localization error obtained from the CNN models for the first scenario. The dashed lines represent the overall density for the single-sensor CNN model trained on CS#3 (Part I) with a y-direction receiving antenna, while the solid lines represent the overall density for the three sensor CNN model.

**Figure 12:**
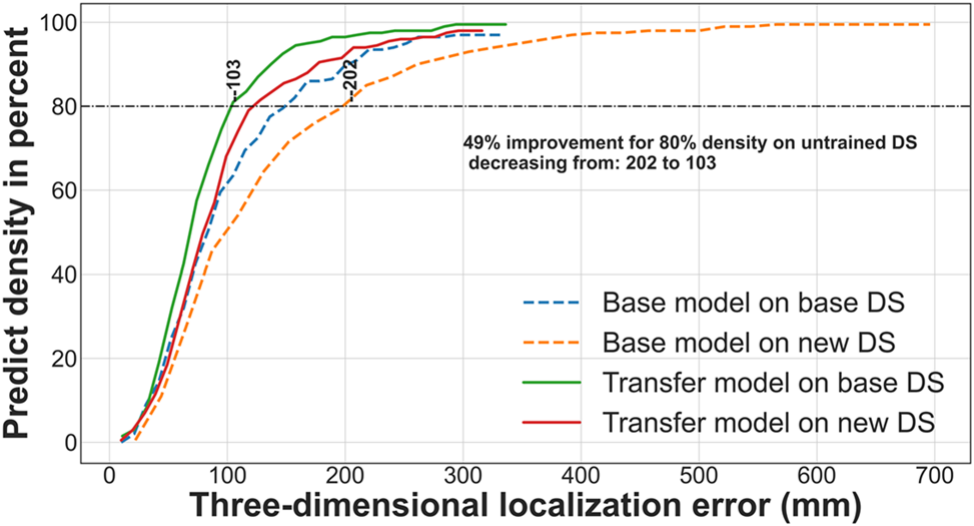
The density of three-dimensional localization error obtained from the CNN models for the second scenario. The dashed lines represent the overall density for the single-sensor CNN model trained on CS#3 (Part II) with a y-direction receiving antenna, while the solid lines represent the overall density for the three-sensor CNN model.

Figures 11 and 12 display the overall density of the three-dimensional localization error obtained from the CNN models on CS#3 (Part I) and (Part II) (refer to the fourth column of Table 3). The dashed lines represent the overall density for the single-sensor pre-trained CNN model, while the solid lines represent the overall density for the three-sensor CNN model. According to these figures, using three sensors leads to a more robust model while evaluating unseen data for 80% density regarding transfer model performance on the base model’s dataset (DS) and base model performance on the transfer model’s DS.

### Effect of the cavity shape and size

In the previous case studies, the localization accuracy in the x direction was observed to be higher than in the other directions. The only difference between the directions in the procedure is in the lengths of the transformer tank sides. Specifically, the x direction is longer than the others. Two experiments were conducted in CS#4 and CS#5 changing the tank dimensions and using the same single-sensor CNN model used in the preceding case studies to examine the impact of the shape and size of the cavity (see Table 1).

In the first experiment, CS#4 was used to determine the relationship between the accuracy of the predicted PD localization in all coordinates and the shape of the cavity. Since the cavity in this dataset has dimensions of 1000 × 1000 × 500 mm^3^, (compare to the 500 × 1000 × 500 mm^3^ dimensions of the previous case studies), it is expected that the accuracy in the x and y directions will be approximately the same. This is indeed the case, as indicated in Table 4.

**Table 4:** RMSE in mm and correlation coefficient (R) for the CNN method for CS#4 and CS#5.

Case study	Direction	Accuracy		Case study	Direction	Accuracy	
		R	RMSE (mm)			R	RMSE (mm)
CS#4 X direction	x	0.8755	109.1582	CS#5 X direction	x	0.8392	242.6377
	y	0.8685			y	0.6205	
	z	0.8550			z	0.6104	
CS#4 Y direction	x	0.8378	121.2639	CS#5 Y direction	x	0.9141	190.1929
	y	0.8382			y	0.7397	
	z	0.8482			z	0.8008	
CS#4 Z direction	x	0.6192	174.0885	CS#5 Z direction	x	0.9227	199.3289
	y	0.6368			y	0.7285	
	z	0.6651			z	0.6593	

In the second experiment, CS#5, the cavity size was increased by a factor of 2 compared to case study CS#3, resulting in dimensions of 2000 × 1000 × 1000 mm^3^. Comparatively, the accuracy remains approximately constant compared to the CS#3 (refer to the fourth column of Table 2 and fourth column of Table 4).

## Conclusions

In this study, a DL-based approach was presented for the 3D localization of PDs within the transformer tanks. Four models were examined, namely BPNN, CNN, SVR, and XGBoost, which were selected based on their frequency in recent related articles and their previous success in localization tasks. Five case studies were considered for this study, each encompassing various conditions such as the maximum and minimum frequency content of the PD signals, antenna and PD source polarization, and the size of the transformer tank. These case studies were generated through Monte Carlo simulations. The models were developed using the Python language on a GPU processor to enhance the computational process.

CNN showed significant accuracy compared to the other models, with an average correlation coefficient of 0.98 and 0.86 for all dimensions in the case studies CS#2 (maximum frequency of 3 GHz) and CS#3 (random maximum frequency in the y-direction), respectively. In the former case study, 99.2% of the localizations had an error of less than 13.3 cm, and in the latter, 88% had an error of less than 17 cm. However, CNN still exhibited limitations in practical robustness. To address this problem, a three-sensor CNN model was introduced, which demonstrated a 26% improvement in robustness compared to the single sensor model, as well as at least a 22% improvement in accuracy. The accuracy of the models is related to the size of the cavity; however, there is no simple relationship. Based on the experiments, the models performed much better in a cavity with two equal dimensions.

The most challenging aspect of implementing this research in practice is collecting enough signals from different types of real power transformers in various locations where PD sources occur. In future work, the proposed method will be applied to a practical power transformer using signals received by a single antenna inside the transformer tank under real-world conditions.

### Supplementary Information

Supplementary Information.

## Data Availability

The datasets generated and analyzed during the current study, as well as the source codes and all computed results, figures, and other related materials, are available in the “Partial_Discharge” repository at github.com/Farzinkh/Partial_Discharge.
