# Supplementary material for: Partial discharge localization in power transformer tanks using machine learning methods
Source: Sci Rep. 2024 May 23;14:11785. doi: 10.1038/s41598-024-62527-9 (PMC11116502; doi:10.1038/s41598-024-62527-9)
Supplement: Supplementary file 1 — Supplementary Information. [file 41598_2024_62527_MOESM1_ESM.docx]

**Supplementary Information**

**Partial Discharge Localization in Power Transformer Tanks Using Machine Learning Methods**

**Farzin Khodaveisi** ^1^**, Hamidreza Karami** ^2^ **^,^ *, Matin Zarei Karimpour** ^3^**, Marcos Rubinstein** ^2^ **and Farhad Rachidi** ^4^

^1^ Department of Electrical Engineering, Bu-Ali Sina University, Hamedan, Iran;

^2^ University of Applied Sciences of Western Switzerland (HES-SO), 1400 Yverdon-les-Bains, Switzerland;

^3^ Electrical Engineering Department, Hamedan University of Technology, Hamedan, Iran;

^4^ Electromagnetic Compatibility Laboratory, Swiss Federal Institute of Technology (EPFL), 1015 Lausanne, Switzerland;

***** Correspondence: [Hamidreza.karami@heig-vd.ch](mailto:Hamidreza.karami@heig-vd.ch); Tel.: +41 787186747

**Contents of this file**

The supplementary information contains 1 figure and 1 table.

Tables

**Table S1.** Summary of conventional ML based PD diagnostics.

| **Index** | **Application** | **objective** | **Methodology** | **Reported Accuracy** |
| --- | --- | --- | --- | --- |
| ^1^ | GIS | Detection | Domain Adaptive Deep Transfer Learning | 98.90% |
| ^2^ | GIS | (PR) Classification | MixNet Deep Learning Model | 99.10% |
| ^3^ | GIS | (PR) Classification | Deep residual CNN +  multi-information ensemble learning | 97.50% |
| ^4^ | XLPE cable | Classification | CNN based transfer learning | Clean data 98.4% |
| ^5^ | Power line | Detection | temporal CNN | 0.97% |
| ^6^ | Elec. equip. | Detection | k-means + OneClassSVM/ Isolation Forest/Local Outlier Factor +CNN | --- |
| ^7^ | Elec. equip. | Detection | CNN based transfer learning | 97.40% |
| ^8^ | cable | Detection | SVM/NN/ensemble tree | 92% for ensemble tree |
| ^9^ | GIS | (PR) Classification | 1DCNN and domain adversarial  transfer learning | 92.77% DATL dataset IV |
| ^10^ | Transformer | Classification | CNN | 100% |
| ^11^ | Elec. equip. | Clustering | k-means/GM/BM | --- |
| ^12^ | Insulated conductors | Detection | CNN‑LSTM of attention mechanisms | 93.89% |
| ^13^ | GIS | (PR) Classification | differentiable neural network | 97.62% |
| ^14^ | Elec. equip. | Classification | SVM (Coarse Gaussian) | 100% |
| ^15^ | GIS | (PR) Classification | Cluster graph convolutional network | 99.58% |

| **Index** | **Application** | **objective** | **Methodology** | **Reported Accuracy** |
| --- | --- | --- | --- | --- |
| ^16^ | Transformer | Detection | PCA,ET + SVM | 96% |
| ^17^ | Elec. equip. | (PR) Classification | SVM-Gaussian | 88.33% |
| ^18^ | GIS | (PR) Classification | Capsule deep graph convolutional network | 97.57% |
| ^19^ | GIS | Classification | DBN | 84% for Floating electrode |
| ^20^ | GIS | (PR) Classification | CNN-LSTM | 97.90% |
| ^21^ | Transformer | (PR) Classification | MobileNet | 98.71% |
| ^22^ | GIL | Localization | ANFIS | 19.69 (mm) for VS |
| ^23^ | GIL | Localization | NNI + ECOC‐MLP‐SVM | ---_ |
| ^24^ | Transformer | Detection | Ultra-micro-CNN | 94.4% with BN for spectrum D |
| ^25^ | GIL | Localization | Bagging-KELM/KELM /BPNN | 0.93 (cm) Bagging-KELM for K3 |
| ^26^ | Power line | Recognition | HHT | 98.5 for all 10 features |
| ^27^ | GIS | (PR) Classification | CNN/SMOTE+SVM | 91.25% for CVAE+CNN |
| ^28^ | GIS | (PR) Classification | IFCNN | 95.58% |
| ^29^ | Elec. equip. | Detection | AdaBoost | 100% |
| ^30^ | Power cable | Localization | CRNN | 94% to 100% with FR |
| ^31^ | Transformer | (PR) Classification | DL based on Parallel feature domain | 94.3% for T2 |
| ^32^ | Elec. equip. | Classification | ResNet | 87.00% |
| ^33^ | XLPE cable | Detection | DWT + CNN | 97.50% |

| **Index** | **Application** | **objective** | **Methodology** | **Reported Accuracy** |
| --- | --- | --- | --- | --- |
| ^34^ | Transformer | Detection | SVM/KNN/DECISION TREE/ENSEMBLER/ADABOOST | 88.6% for AdaBoost |
| ^35^ | Transformer | Classification | HSVD-HG | 99.2% after HP |
| ^36^ | Ceramic insulators | Classification | kNN/Naive Bayes/Decision tree/SVM | 90.6% for SVM |
| ^37^ | Elec. equip. | Localization | TOA | 0.3% of the true location of the PD source with negligible error |
| ^38^ | Elec. equip. | Detection | BI-LSTM | --- |
| ^39^ | Transformer | Localization | EEMD + PE- IED + F-PCA + ADBSCAN | --- |
| ^40^ | XLPE cable | Detection | LSTM | 88.57% |
| ^41^ | GIS | Detection | DT/DA/NB/KNN/EN | 97.1% for EN |
| ^42^ | GIS | Detection | MBML | 93.17% |
| ^43^ | Transformer | (PR) Classification | Multifrequency fiber-optic F–P ultrasound Sensing Array, AOK, TFR | 98.00% |
| ^44^ | Elec. equip. | (PR) Classification | DCNN + SVM | 95.35% or NL SVM Sigmoidal |
| ^45^ | GIS | Classification | Random forest/KNN | 95% for RF |
| ^46^ | Elec. equip. | Localization | GPC/BP/SVM | 2.51 (m) Avg error for GPC |
| ^47^ | Oil tank | Localization | FLTS | 0.02 (m) Error |

Figures


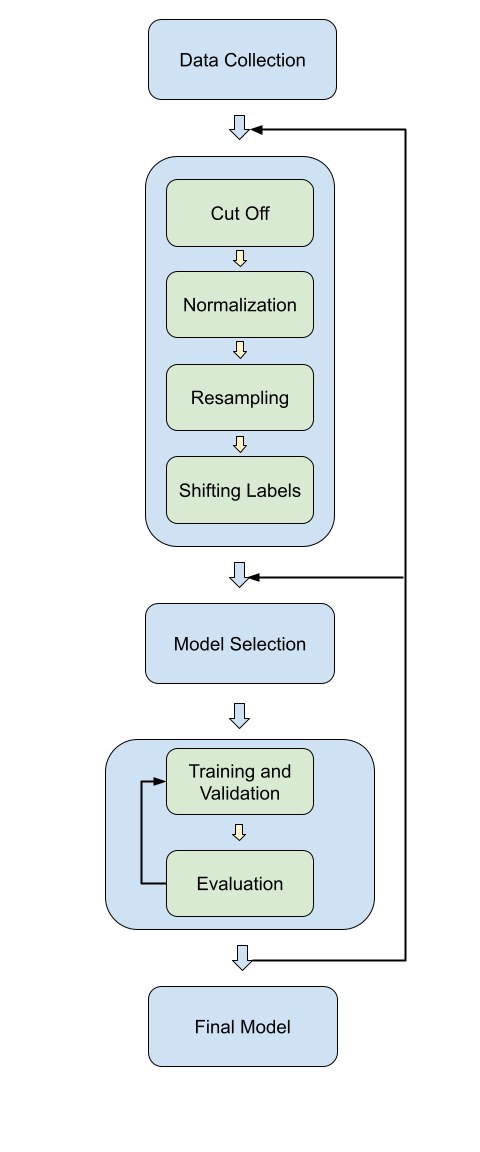


Fig. S1 A flowchart depicting the ML and DL-based approaches proposed in the paper.

The flowchart depicting the ML and DL-based approaches proposed in the paper is presented in Figure S1. The initial step involves data collection, which is simulated using CST-MWS software. Once the data is collected, it needs to undergo preprocessing before being fed into the models. During preprocessing, the data is initially trimmed and then normalized to fall within the range of 1 to -1. Following normalization, the data is resampled to consist of 400 samples. Given that the labels span from negative to positive numbers, representing the PD source’s location within the cavity along the x-, y-, and z-axes, the location labels’ origin is shifted to ensure all labels are positive.

1. Wang, Y. *et al.* A Domain Adaptive Deep Transfer Learning Method for Gas-Insulated Switchgear Partial Discharge Diagnosis. *IEEE Trans. Power Deliv.* **37**, (2022).

2. Wang, Y., Yan, J., Yang, Z., Zhao, Y. & Liu, T. Optimizing GIS partial discharge pattern recognition in the ubiquitous power internet of things context: A MixNet deep learning model. *Int. J. Electr. Power Energy Syst.* **125**, (2021).

3. Jing, Q., Yan, J., Lu, L., Xu, Y. & Yang, F. A Novel Method for Pattern Recognition of GIS Partial Discharge via Multi-Information Ensemble Learning. *Entropy* vol. 24 (2022).

4. Raymond, W. J. K., Xin, C. W., Kin, L. W. & Illias, H. A. Noise invariant partial discharge classification based on convolutional neural network. *Meas. J. Int. Meas. Confed.* **177**, (2021).

5. Michau, G., Hsu, C. C. & Fink, O. Interpretable detection of partial discharge in power lines with deep learning. *Sensors* **21**, (2021).

6. Florkowski, M. Anomaly detection, trend evolution, and feature extraction in partial discharge patterns. *Energies* **14**, (2021).

7. Kim, J. & Kim, K. Il. Partial discharge online detection for long-term operational sustainability of on-site low voltage distribution network using CNN transfer learning. *Sustain.* **13**, (2021).

8. Abu-Rub, O. H., Khan, Q., Refaat, S. S. & Nounou, H. Cable Insulation Fault Identification Using Partial Discharge Patterns Analysis. *IEEE Can. J. Electr. Comput. Eng.* **45**, (2021).

9. Wang, Y., Yan, J., Yang, Z., Wang, J. & Geng, Y. A novel 1DCNN and domain adversarial transfer strategy for small sample GIS partial discharge pattern recognition. *Meas. Sci. Technol.* **32**, (2021).

10. Mantach, S., Ashraf, A., Janani, H. & Kordi, B. A convolutional neural network-based model for multi-source and single-source partial discharge pattern classification using only single-source training set. *Energies* **14**, (2021).

11. Kaimal, L. & Kulkarni, R. Automatic Estimation of Multiplicity in Partial Discharge Sources Using Machine Learning Techniques BT - Advances in Electrical and Computer Technologies. in (eds. Sengodan, T., Murugappan, M. & Misra, S.) 519–534 (Springer Nature Singapore, 2022).

12. Li, Z., Qu, N., Li, X., Zuo, J. & Yin, Y. Partial discharge detection of insulated conductors based on CNN-LSTM of attention mechanisms. *J. Power Electron.* **21**, (2021).

13. Jing, Q., Yan, J., Wang, Y., He, R. & Lu, L. A Novel Differentiable Neural Network Architecture Automatic Search Method for GIS Partial Discharge Pattern Recognition. *SSRN Electron. J.* (2022) doi:10.2139/ssrn.4031281.

14. Kumar, H., Shafiq, M., Hussain, G. A. & Kauhaniemi, K. Comparison of Machine Learning Algorithms for Classification of Partial Discharge Signals in Medium Voltage Components. in *Proceedings of 2021 IEEE PES Innovative Smart Grid Technologies Europe: Smart Grids: Toward a Carbon-Free Future, ISGT Europe 2021* (2021). doi:10.1109/ISGTEurope52324.2021.9639923.

15. Tian, J., Song, H., Sheng, G. & Jiang, X. Knowledge-Driven Recognition Methodology of Partial Discharge Patterns in GIS. *IEEE Trans. Power Deliv.* **37**, (2022).

16. Jiang, J. *et al.* Partial Discharge Detection and Diagnosis of Transformer Bushing Based on UHF Method. *IEEE Sens. J.* **21**, (2021).

17. Yao, R., Li, J., Hui, M., Bai, L. & Wu, Q. Pattern recognition for partial discharge using multi-feature combination adaptive boost classification model. *IEEE Access* **9**, (2021).

18. Wang, Y. *et al.* GIS partial discharge pattern recognition via a novel capsule deep graph convolutional network. *IET Gener. Transm. Distrib.* **16**, 2903–2912 (2022).

19. Li, J. *et al.* Intelligent diagnosis and recognition method of GIS partial discharge data map based on deep learning. in *2021 Power System and Green Energy Conference (PSGEC)* 253–256 (IEEE, 2021).

20. Liu, T., Yan, J., Wang, Y., Xu, Y. & Zhao, Y. Gis partial discharge pattern recognition based on a novel convolutional neural networks and long short‐term memory. *Entropy* **23**, (2021).

21. Sun, Y. *et al.* Partial discharge pattern recognition of transformers based on mobilenets convolutional neural network. *Appl. Sci.* **11**, (2021).

22. Zang, Y. *et al.* A Novel Optical Localization Method for Partial Discharge Source Using ANFIS Virtual Sensors and Simulation Fingerprint in GIL. *IEEE Trans. Instrum. Meas.* **70**, (2021).

23. Zang, Y. *et al.* Method of GIL partial discharge localization based on natural neighbour interpolation and ECOC-MLP-SVM using optical simulation technology. *High Volt.* **6**, (2021).

24. Liu, Y., Hu, M., Dai, Q., Le, H. & Liu, Y. Online recognition method of partial discharge pattern for transformer bushings based on small sample ultra-micro-CNN network. *AIP Adv.* **11**, (2021).

25. Zang, Y. *et al.* A novel partial discharge localization method for GIL based on the 3D optical signal irradiance fingerprint and bagging-KELM. *IET Gener. Transm. Distrib.* **15**, 2240–2249 (2021).

26. Wang, Y., Chiang, H. & Dong, N. Power-Line Partial Discharge Recognition with Hilbert–Huang Transform Features. *Energies* **15**, 6521 (2022).

27. Jing, Q., Yan, J. & Wang, Y. A novel partial discharge pattern recognition for GIS with unbalanced sample based on conditional variational autoencoder. (2022).

28. Zheng, J., Chen, Z., Wang, Q., Qiang, H. & Xu, W. GIS Partial Discharge Pattern Recognition Based on Time-Frequency Features and Improved Convolutional Neural Network. *Energies* **15**, 7372 (2022).

29. Lin, Y. *et al.* Partial discharge diagnosis algorithm for multi-source ultrasound detection based on time series integration. in *Proceedings - 2021 6th Asia Conference on Power and Electrical Engineering, ACPEE 2021* (2021). doi:10.1109/ACPEE51499.2021.9437002.

30. Yeo, J. *et al.* Localisation of Partial Discharge in Power Cables Through Multi-Output Convolutional Recurrent Neural Network and Feature Extraction. *IEEE Trans. Power Deliv.* (2022).

31. Xu, Y., Xia, H., Xie, S. & Lu, M. The pattern recognition of multisource partial discharge in transformers based on parallel feature domain. *IET Sci. Meas. Technol.* **15**, 163–173 (2021).

32. Borghei, M. & Ghassemi, M. A deep learning approach for discrimination of single- And multi-source corona discharges. *IEEE Trans. Plasma Sci.* **49**, (2021).

33. Wang, M. H., Lu, S. Der & Liao, R. M. Fault Diagnosis for Power Cables Based on Convolutional Neural Network with Chaotic System and Discrete Wavelet Transform. *IEEE Trans. Power Deliv.* **37**, (2022).

34. Balaraman, S. *et al.* Fault Diagnosis and Asset Management of Power Transformer Using Adaptive Boost Machine Learning Algorithm. *IOP Conf. Ser. Mater. Sci. Eng.* **1055**, (2021).

35. Govindarajan, S., Ragavan, V., El-Hag, A., Krithivasan, K. & Subbaiah, J. Development of hankel singular-hypergraph feature extraction technique for acoustic partial discharge pattern classification. *Energies* **14**, (2021).

36. Ma, D., Jin, L., He, J. & Gao, K. Classification of partial discharge severities of ceramic insulators based on texture analysis of UV pulses. *High Volt.* **6**, (2021).

37. Ghanakota, K. C., Ramanujam, S. & Arunachalam, K. Numerical Analysis of Partial Discharge Source Localization Using Time of Arrival Measurements and Nonlinear Least Squares Search. in *Lecture Notes in Mechanical Engineering* (2021). doi:10.1007/978-981-16-0186-6_26.

38. Zhou, S. & Wang, Y. Partial discharge fault diagnosis based on optimized BI-LSTM. in *7th International Symposium on Advances in Electrical, Electronics, and Computer Engineering* vol. 12294 122–128 (SPIE, 2022).

39. Govindarajan, S., Natarajan, M., Ardila-Rey, J. A. & Venkatraman, S. Partial Discharge Location Identification Using Permutation Entropy Based Instantaneous Energy Features. *IEEE Trans. Instrum. Meas.* **70**, (2021).

40. Xu, N., Gooi, H. B., Wang, L., Zheng, Y. & Yang, J. Partial Discharge Detection Based on Long Short-Term Memory Neural Network Classifier with Efficient Feature Extraction Methods. in *Proceedings of the Energy Conversion Congress and Exposition - Asia, ECCE Asia 2021* (2021). doi:10.1109/ECCE-Asia49820.2021.9479331.

41. Mansour, D.-E. A., Taha, I. B. M., Farade, R. A. & Wahab, N. I. B. A. Partial Discharge Diagnosis in GIS based on Pulse Sequence Features and Optimized Machine Learning Classification Techniques. *Electr. Power Syst. Res.* **211**, 108162 (2022).

42. Wang, Y. *et al.* Novel metric-based meta-learning model for few-shot diagnosis of partial discharge in a gas-insulated switchgear. *ISA Trans.* (2022).

43. Zhang, Z. *et al.* Partial Discharge Pattern Recognition Based on a Multifrequency F–P Sensing Array, AOK Time–Frequency Representation, and Deep Learning. *IEEE Trans. Dielectr. Electr. Insul.* **29**, 1701–1710 (2022).

44. Aruna Devi, I., Maheswari, R. V. & Rajesh, R. Recognition of Fused Partial Discharge Patterns in High Voltage Insulation Systems: A Hybrid DCNN and SVM Based Approach. *IETE J. Res.* (2022) doi:10.1080/03772063.2022.2038702.

45. Jayaganthan, S., Guvvala, N. & Ramanujam, S. Identification and Classification of Incipient Discharges in GIS Adopting Machine Learning Techniques. in *Proceedings of the IEEE International Conference on Properties and Applications of Dielectric Materials* vols 2021-July (2021).

46. Zhang, C., Liu, G., Chen, D., Xu, T. & Gong, P. A Machine Learning Approach for UHF Partial Discharge Localization. in *2022 7th Asia Conference on Power and Electrical Engineering (ACPEE)* 2219–2223 (IEEE, 2022).

47. Hashim, A. H. M. *et al.* Partial discharge localization in oil through acoustic emission technique utilizing fuzzy logic. *IEEE Trans. Dielectr. Electr. Insul.* **29**, 623–630 (2022).
